# Second-Order Phase Transition in Counter-Rotating Taylor–Couette Flow Experiment

**DOI:** 10.3390/e23010058

**Published:** 2020-12-31

**Authors:** Kerstin Avila, Björn Hof

**Affiliations:** 1 Faculty of Production Engineering, University of Bremen, Badgasteiner Strasse 1, 28359 Bremen, Germany; 2 Leibniz Institute for Materials Engineering IWT, Badgasteiner Strasse 3, 28359 Bremen, Germany; 3 Institute of Science and Technology Austria, Am Campus 1, 3400 Klosterneuburg, Austria; bhof@ist.ac.at

**Keywords:** phase transition, Couette flow, lifetimes

## Abstract

In many basic shear flows, such as pipe, Couette, and channel flow, turbulence does not arise from an instability of the laminar state, and both dynamical states co-exist. With decreasing flow speed (i.e., decreasing Reynolds number) the fraction of fluid in laminar motion increases while turbulence recedes and eventually the entire flow relaminarizes. The first step towards understanding the nature of this transition is to determine if the phase change is of either first or second order. In the former case, the turbulent fraction would drop discontinuously to zero as the Reynolds number decreases while in the latter the process would be continuous. For Couette flow, the flow between two parallel plates, earlier studies suggest a discontinuous scenario. In the present study we realize a Couette flow between two concentric cylinders which allows studies to be carried out in large aspect ratios and for extensive observation times. The presented measurements show that the transition in this circular Couette geometry is continuous suggesting that former studies were limited by finite size effects. A further characterization of this transition, in particular its relation to the directed percolation universality class, requires even larger system sizes than presently available.

## 1. Introduction

In shear flows, turbulence tends to first appear in spatially localized patches that are interspersed by quiescent, laminar regions, a phenomenon commonly referred to as spatio-temporal intermittency. The resulting flow pattern chaotically changes in time and unless the entire flow relaminarises, it never settles to a steady state. One of the earliest reports of laminar turbulent intermittency dates back to Osborne Reynolds and his study of pipe flow [1]. The corresponding turbulent “flashes” or “puffs” are quasi-one-dimensional, meaning that they tend to fill out the radial-azimuthal pipe cross-section, whilst being localized in the streamwise direction [2]. Puffs have a well defined mean length; however, their spacing and hence the size of the laminar gaps is irregular and continuously changes. The resulting overall flow pattern can be accurately modeled as one dimensional [3]. In flows that are extended in two spatial dimensions, but strongly confined in the third (such as channel and Couette flows), turbulence forms elongated stripes [4,5,6,7]. Here turbulence fills the wall normal gap and is localized in the extended streamwise and spanwise directions. The resulting laminar-turbulent intermittent stripe pattern can be regarded as quasi-two-dimensional.

In quasi-one- and two-dimensional cases alike, individual patches of turbulence have finite lifetimes and eventually decay. Early propositions that individual turbulent patches (or turbulence in small domains) become sustained at a critical point [8,9,10,11] turned out to be incorrect. Despite their often long lifetimes individual patches remain transient and eventually decay following a memoryless process [12,13,14,15,16]. In line with other contact processes such as directed percolation [17] and coupled map lattices [18,19] and as pointed out in the context of shear flows [20,21] spatial proliferation of active sites can give rise to a phase transition to sustained turbulence. Specifically it has been demonstrated for pipe flow [22,23] that turbulence becomes sustained via a contact process where individual localized patches remain transient but can seed new patches before they decay. Also puff splitting has been found to be a memoryless process, a circumstance that allowed to determine the critical point for pipe flow as the balance point between lifetimes and splitting rates [23].

A key remaining question regarding the onset of turbulence, for both one-dimensional and two-dimensional cases alike, is whether the transition is of first or second order (in the context of contact processes and phase transitions in statistical physics, see [24]). In a second-order phase transition, the turbulent fraction decreases continuously to zero as the Reynolds number is decreased toward the critical point, whereas in a first-order phase transition the turbulent fraction jumps from a finite value to zero at the critical point. Hence first-order transitions are referred to as discontinuous and second-order transitions as continuous. In both cases, however, the laminar flow is linearly stable and because of the hysteresis the flow must be initialized with turbulence to measure the transition. While for pipe flow the transition is presumed continuous [3], this so far could not be shown explicitly due to the excessive time scales that prohibit to reach a statistical steady state sufficiently close to the critical point [25]. In a circular Couette experiment of large azimuthal and small axial aspect ratio, where flow patterns like in pipe flow can only evolve in one spatial dimension, the transition has been shown to be continuous [26] and to fall into the directed percolation (DP) universality class.

In an earlier study Bottin and Chatté [8] characterized the transition to turbulence in an experimental study of planar Couette flow in a moderately large aspect-ratio (190*d* × 35*d* in the streamwise and spanwise direction, where *d* is the gap). In this two dimensional setting, the turbulent fraction was about 30% close to the onset of sustained turbulence and dropped dramatically to zero (laminar flow) as the Reynolds number was reduced. The authors suggested that the onset of turbulence in plane Couette flow corresponds to first-order phase transition. Duguet et al. [27] did direct numerical simulations of a larger system (400*d* × 178*d*), but with substantially shorter observation times ($2\times 10^{4}$ advective time units), and reported similar results. More recently, Chantry et al. [28] examined numerically the onset of turbulence in Waleffe flow. In contrast to Couette flow, in this case stress-free boundary conditions are applied at the walls and the flow is driven by a sinusoidal body force. The choice of boundary conditions greatly reduces computational cost and allowed direct numerical simulations of a very large aspect-ratio system (1280*d* × 1280*d*) for very long observation times (exceeding $2\times 10^{6}$ advective time units). Their simulations compellingly show that transition in this simple model system falls in the universality class of two-dimensional directed percolation. While suggestive, it nevertheless remains unclear if for quasi-two-dimensional Couette type flows the transition is either of first or second order. For a recent review of the flow patterns and dynamics of wall-bounded flows extended in two directions, see Tuckerman et al. [7].

**Figure 1:**
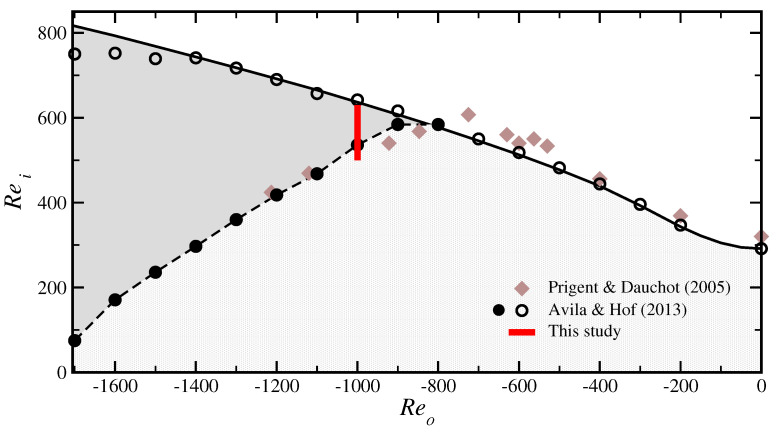
Stability diagram of counter-rotating Taylor–Couette flow with radius ratio η=0.98 and stationary lids, $Re_{\mathrm{lids}}=0$. The solid line shows the linear stability boundary in the infinite–cylinder case. As the the Reynolds number of the outer cylinder ($Re_{o}$) is decreased, the linear instability of the laminar, circular Couette flow is shifted to higher Reynolds number of the inner cylinder ($Re_{i}$). The empty symbols denote our experimental measurements of the onset of instability, obtained by increasing $Re_{i}$ at fixed $Re_{o}$, which we reported previously in [35]. Subcritical turbulence in the form of turbulent stripes and spots is found in the shaded region starting at $Re_{o}\approx -800$; the full symbols mark the relaminarization of subcritical turbulence and were obtained by decreasing $Re_{i}$ at fixed $Re_{o}$, in order to detect hysteresis. In this paper, the subcritical transition at $Re_{o}=-1000$ is analyzed in more detail (statistically) to shed light on the nature of this phase transition to turbulence. For comparison the data of Prigent et al. and coworkers (diamonds) [5,36,37] for a similar radius ratio η are shown, indicating the sensitivity of the flow to finite amplitude perturbations.

In Taylor–Couette flow between two counter-rotating cylinders, the flow dynamics is qualitatively similar to plane Couette flows [4,5,16,29,30] provided that the laminar velocity profile is linearly stable. Indeed, in the narrow-gap limit $\eta =r_{i}/r_{o}\to 1$, where $r_{i}$ and $r_{o}$ are the radii of the inner and outer cylinders, Taylor–Couette flow turns into rotating plane Couette flow [31]. For fully turbulent flows, the dynamics of Taylor–Couette flow converges to that of rotating plane Couette flow already for moderately small gaps η≥0.9 [32]. By contrast, the dynamics of transition for exactly counter-rotating cylinders is alike that of plane Couette flow only for very narrow gaps η≥0.993 [33]; for larger gaps the linear centrifugal (Rayleigh) instability occurs at lower Reynolds number than the subcritical transition. We note that a new linear instability of counter-rotating Taylor–Couette flow was recently discovered [34], however this instability occurs for extremely high Reynolds numbers (for η>0.9, $|Re_{o}|>10^{8}$, where $Re_{o}$ is the Reynolds number of the outer cylinder) and disappears in the narrow gap limit. This instability is far away in parameter space of the experiments performed here, with $Re_{o}=O\left(10^{3}\right)$. In Figure 1 we show a regime diagram of counter-rotating Taylor–Couette flow of radius ratio of η=0.98. In the infinite-cylinders case, the onset of Taylor vortices is at $Re_{i}=292$ when the outer cylinder does not rotate ($Re_{o}=0$). For increasing counter-rotation of the outer cylinder, the linear stability threshold rises to higher $Re_{i}$ and the stability boundary previously measured with our experimental setup [35] is in excellent quantitative agreement with the linear stability analysis of the infinite-cylinder case (solid line in Figure 1), and to a lesser extent also with the experimental measurements of Prigent and Dauchot [36]. For moderately strong counter-rotation ($Re_{o}<-800$), turbulence can be triggered via finite amplitude perturbations well below the linear instability. Such perturbations occurred naturally in the experimental setup of Prigent and Dauchot [36], whereas in our setup a progressively growing band of hysteresis between the onset of linear instability and the decay of sub-critical turbulence can be observed.

In Avila and Hof [35], the critical Reynolds number for self-sustained turbulence was measured by quasi-statically decreasing $Re_{i}$ in steps of 1 min. This measurement procedure is suited to obtain a rough estimate of the transition border, but does not take into account the stochastic nature of turbulence decay. Measurements of the lifetimes statistics are required here, as previously performed in a small aspect ratio Taylor–Couette flow (55*d* × 34*d*) [16]. Compared to all previous quasi-two dimensional Couette or Taylor–Couette experiments, our system’s streamwise-spanwise area is at least 12 times larger (311*d* × 263*d*), see Table 1. This allows us to study the nature of the turbulence transition with a reduced influence of finite-size effects. We show that lifetimes are exponentially distributed below the critical point and that the increase of the turbulent fraction beyond the critical point is continuous and therefore of second order.

## 2. Experimental Methods

The Taylor–Couette experiment used in this study consists of two concentric cylinders with radii $r_{i}=\left(110.25\pm 0.025\right)$ mm and $r_{o}=\left(112.53\pm 0.05\right)$ mm leading to a radius ratio η=0.98 and an azimuthal length of 311 gap width $d=r_{o}-r_{i}=2.28$ mm. The Reynolds number of the inner (outer) cylinder with angular velocity $\omega_{i}$ ($\omega_{o}$) is defined as $Re_{i}=\omega_{i}r_{i}d/\nu$ ($Re_{o}=\omega_{o}r_{o}d/\nu$), where ν is the kinematic viscosity of the working fluid. The azimuthal direction is in our system the streamwise direction and is naturally periodic (in contrast to Couette flow experiments); this eliminates end effects in the streamwise direction. The axial (spanwise) direction is bounded by the axial lids and has a length of 263*d*. The lids can be rotated independently of the cylinders. Their Reynolds number is based on the radius of the outer cylinder ($Re_{\mathrm{lid}}=\omega_{\mathrm{lid}}r_{o}d/\nu$). In many Taylor–Couette experiments, the lids are attached to the outer cylinder to reduce the Ekman pumping, see, e.g., [16,39,40,41]. For example, spiral patterns are less influenced by the axial lids, when the lids co-rotate with the outer cylinder, than when they are stationary [42]. The effect of axial boundary conditions was investigated systematically in experiments [43] and in simulations [44], that showed that rotating the lids at angular speeds between the inner and outer cylinder leads to laminar flows closest to circular Couette flow. For our setup and selected parameter regime, $Re_{\mathrm{lids}}=-800$ minimized end effects, but the spatio-temporal dynamics was identical for lids attached to the outer cylinder $Re_{\mathrm{lids}}=-1000$, and for stationary lids $Re_{\mathrm{lids}}=0$, because of the large height-to-gap aspect ratio. Rotating the lids merely led to a slight stabilization of the laminar flow and hence to a small shift of the onset of turbulence to slightly higher $Re_{i}$.

The viscosity of the working fluid silicone oil was determined by measuring the onset of Taylor vortices for stationary outer cylinder as the inner cylinder rotation was increased. Specifically, the value of the viscosity was selected to match the critical inner Reynolds number obtained with a linear stability analysis of laminar, circular Couette flow between infinite cylinders ($Re_{i,c}=292$ at $Re_{o}=0$ ). The accuracy of this method and of our experiment is verified in the excellent agreement obtained with the linear stability results throughout the counter-rotating regime. In particular, the discrepancy is less than 1% in $Re_{i}$ when comparing the experimentally measured and the theoretical stability curves. For the visualization of the flow the working fluid silicone oil was seeded with aluminium platelets.

**Figure 2:**
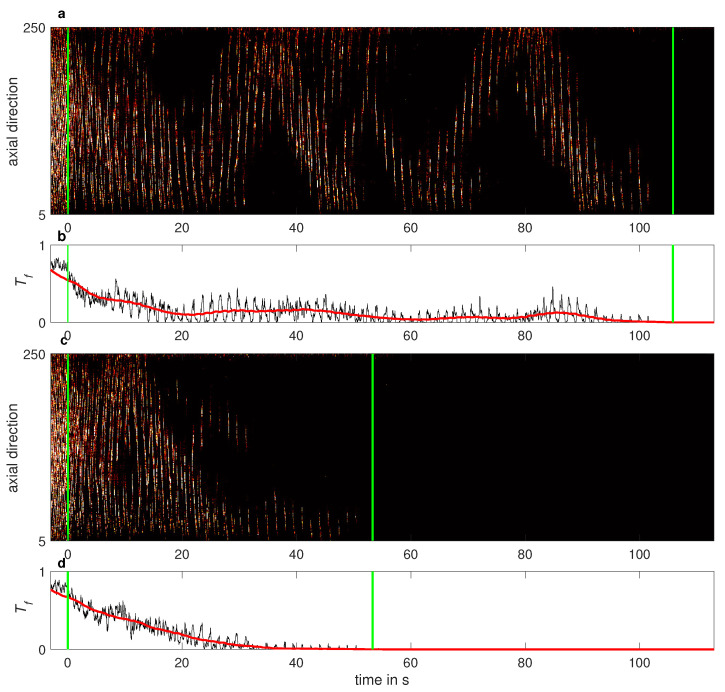
(**a**,**c**) Spati-temporal dynamics of two selected lifetime measurements at $Re_{i}=530$, $Re_{\mathrm{lids}}=-800$. (**b**,**d**) Corresponding instantaneous (black solid line) and averaged (red thick line) turbulent fraction. The average turbulent fraction is calculated in windows of about 9 s (moving-average technique) to illustrate the long-time dynamics and is used to detect the relaminarization of the flow. The left green line marks the time of the reduction in $Re_{i}$ and the right green line the decay of turbulence. The determined lifetime corresponds to the time interval between the two green lines.

**Figure 3:**
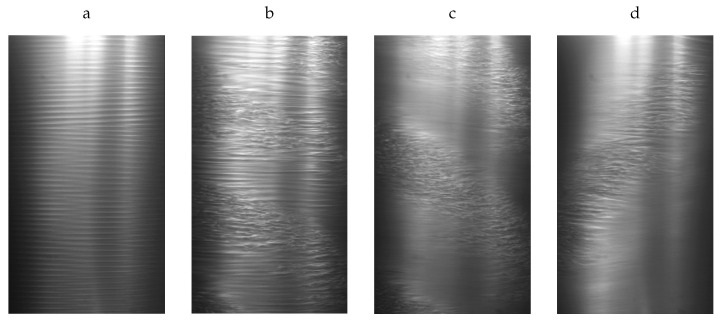
Snapshots of typical flow patterns in counter-rotating Taylor–Couette flow. (**a**) The linear instability arises in the counter-rotating regime in the form of laminar spirals (snapshot taken at $Re_{i}=560$, $Re_{o}=-700$). (**b**) Laminar spirals can coexist with turbulent spots frequently decaying and arising, or they can aligne into stripes, as shown here ($Re_{i}=700$, $Re_{o}=-700$). (**c**) Laminar-turbulent intermittency in the form of subcritical turbulent stripes ($Re_{i}=600,Re_{o}=-1000$). (**d**) For decreasing $Re_{i}$ the regions of laminar flow around the turbulent stripes increase in area ($Re_{i}=540,Re_{o}=-1000$). The field of view corresponds here to about 10% of the total system size area. The axial lids are stationary in all snapshots ($Re_{\mathrm{lids}}=0$). All snapshots were taken in the statistically steady regime.

**Figure 4:**
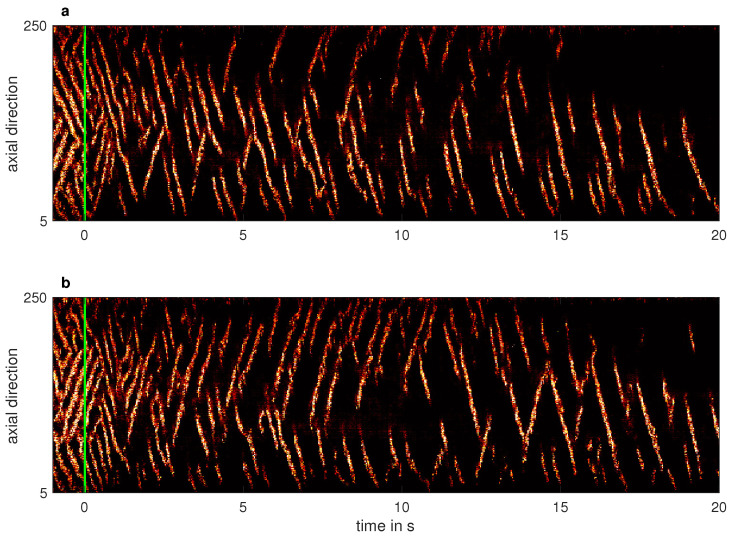
Spatio-temporal dynamics of subcritical turbulence $Re_{\mathrm{lids}}=-800$ (**a**) and $Re_{o}=-1000$ (**b**) following a reduction in $Re_{i}$. Turbulent stripes dominate the dynamics at $Re_{i}=630$, prior to an abrupt reduction to $Re_{i}=530$ (green line). The turbulent fraction decreases immediately after the reduction in $Re_{i}$, but it takes about 20 s for the flow to adjust into a (metastable) statistically steady state. The long-time dynamics of these two cases are is displayed in Figure 2a,b, respectively. The axial direction is in dimensionless units (i.e., normalized with the gap width *d*).

**Figure 5:**
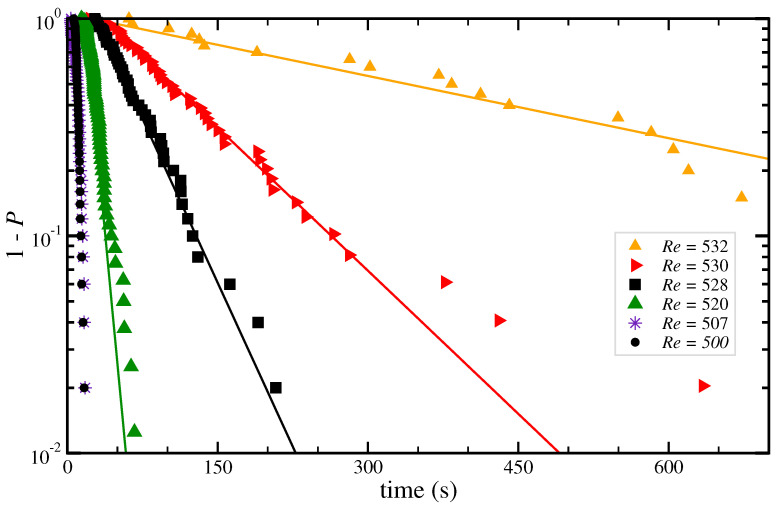
Lifetime statistics at $Re_{o}=-1000$ and $Re_{\mathrm{lids}}=-800$. Shown is the survival probability of turbulence (in a logarithmic scale) as a function of time for several $Re_{i}$, as indicated in the legend. The symbols denote individual measurements, which are sorted in increasing survival time to construct the survival probability function. In all cases, the initial condition was a turbulent flow at $Re_{i}=630$ and the rotation of the cylinder was suddenly changed to the desired $Re_{i}$.

**Figure 6:**
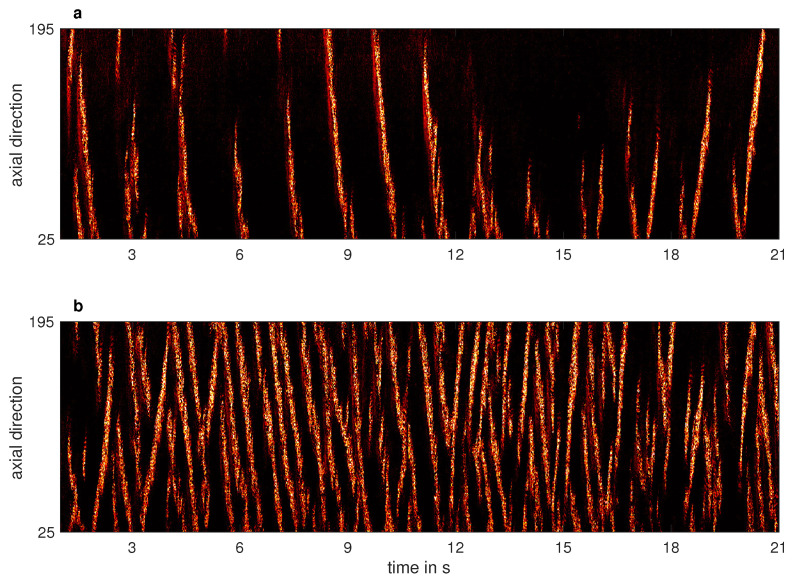
Excerpt of the spatio-temporal dynamics of turbulent stripes ($Re_{o}=-1000$ and $Re_{\mathrm{lids}}=0$) above the critical point for the onset of sustained turbulence. (**a**) $Re_{i}=525$, (**b**) $Re_{i}=532$.

**Figure 7:**
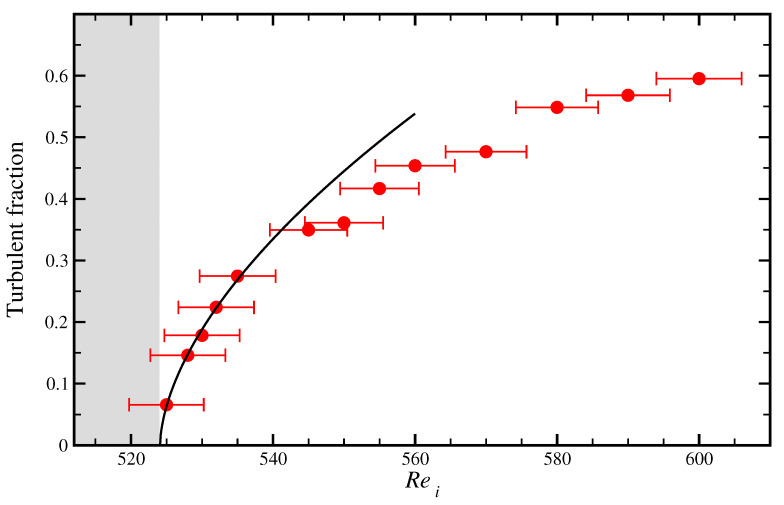
Second-order phase transition in counter-rotating Taylor–Couette flow ($Re_{o}=-1000$ and $Re_{\mathrm{lids}}=0$). The turbulent fraction increases smoothly from a minimum of 7% at $Re_{i}=525$ up to 60% at $Re_{i}=600$. The error bars indicate a 1% deviation in $Re_{i}$, which estimated from the discrepancy between linear stability analysis and experiment in Figure 1. The black line is a fit of the form $T_{f}=a{\left(Re_{i}-Re_{i,c}\right)}^{\beta_{DP}}$, with $\beta_{DP}=0.583$, to the data points in the vicinity of the critical point ($524<Re_{i}<540$). The fit parameters are a=0.0667 and $Re_{i,c}=524.1$. Below the critical point (grey region), turbulence is transient.

The turbulent fraction was determined by analyzing the images from a high speed camera used to monitor the flow. The flow was seeded with highly reflective aluminum platelets (Eckart, Effect Pigments, STAPA WM Chromal V/80 Aluminum) in a concentration below 1% in weight (and volume). In turbulent flows these tracers are randomly oriented and reflect light efficiently. Turbulent flow patches appear therefore brighter than laminar regions. In our image processing code we use this difference in the light intensity to distinguish laminar from turbulent regions by thresholding. The turbulent fraction is calculated at each instant of time in the spatio-temporal diagrams (see, e.g., Figure 2) as axial length covered by turbulent flow in comparison to the axial length of the field of view. Further details of the image analysis are provided in [35]. Videos were typically recorded with 80 Hz and the resolution in the axial direction was 1920 pixels and in the azimuthal direction between 5 and 1080 pixels, from which only 3 were used for the generation of the spatio-temporal diagrams and hence the quantitative analysis. The measurements shown in this paper consist of three independent sets of experiments with slightly different viscosities and different field of views of the camera, each of them optimized for the specific analysis. For the measurements shown in Figure 1 and Figure 3, the working fluid silicon oil has a viscosity of ν=(4.65±0.02)cSt. The field of view of the camera in Figure 3 was (50d×80d), corresponding to about 10% of the total area and was located 46*d* above the lower lid. For the measurements in Figure 4 and Figure 5 the viscosity was ν=(4.55±0.02)cSt and the field of view consisted of a line of 3 pixel width and an axial length of 245*d*, which started 5*d* above the lower lid. For the measurements in Figure 6 and Figure 7 the viscosity was ν=(4.41±0.02)cSt and the field of view was (5*d*× 170*d*) and started 25*d* above the lower lid. More details of the setup and the image analysis and processing that are omitted here can be found in [35].

## 3. Results

The experiments reported in this work were performed at $Re_{o}=-1000$ as indicated by the red line in Figure 1. The dynamics obtained at this selected $Re_{o}$ is representative for the subcritical regime and hence also for other $Re_{o}$.

### 3.1. Lifetimes of Turbulent Stripes and Spots

For the lifetimes measurements, the speed of the lids was held constant at $Re_{\mathrm{lids}}=-800$. The system was perturbed by rapidly accelerating the speed of the inner cylinder to $Re_{i}=630$. This excited at first a linear instability in the form of laminar spirals (see Figure 3a), which quickly evolved into an intermittent pattern of laminar-turbulent stripes (see Figure 3b). The flow was then given sufficient time to reach a statistical steady state pattern. The camera started to record the flow pattern 20 s before $Re_{i}$ was abruptly reduced to one of the six values indicated in the legend of Figure 5. The flow was continuously recorded until it relaminarised. Rather independently of the $Re_{i}$, the turbulent fraction typically dropped monotonically within the first 20 s, as the flow adapted to the new $Re_{i}$. Two examples of the corresponding spatio-temporal dynamics are shown in Figure 4, where the green line indicates the change of the $Re_{i}$ in time. Despite the apparently similar dynamics, the long time behavior of these two cases is very different, leading to different lifetimes, see Figure 2. The complete decay of turbulence was systematically detected by determining the time at which the moving average of the turbulent fraction dropped permanently below a threshold.

During most of the runtime, the axial extent of the turbulent stripes was shorter than the cylinder length. The stripes moved in the axial and azimuthal direction exhibited a rich dynamics, including growth, shrinkage, splitting, merging and decay. Interactions with the axial lids occurred frequently. Specifically, the decay occurred often close to the lids. We thus believe that end effects are likely to influence the turbulent dynamics despite the large axial aspect ratio of our setup.

The probability of survival of turbulence as a function of time is shown in Figure 5. For the two lowest $Re_{i}$ investigated the lifetimes are all shorter than 20 s, which corresponds to the time in which the (averaged) turbulent fraction continuously decreases without developing intrinsic dynamics. Therefore it is unclear wether the corresponding lifetimes are exponentially distributed or not in these two cases. A similar behavior was observed for the decay of puffs in pipe flow at low Re [14] in which the distribution deviated from an exponential one. However, in our measurements the distributions still seems to be exponential and for $Re_{i}>507$ the probability follows $P\left(t\right)=1-\exp[\left(t-t_{0}\right)/\tau$, with the equilibration time $t_{0}$≈ 20 s and τ the characteristic lifetime. This indicates that the decay of turbulence in this regime is a memoryless process, as reported for spatially extended plane Couette flow [8], quasi-one-dimensional [26,38] and moderate aspect-ratio [16] Taylor–Couette flows, and also for pipe flow [14] and quasi-one-dimensional channel flow [45].

### 3.2. Second-Order Phase Transition

In this section we present measurements of the turbulent fraction above the critical point. The measurement procedure was as in the previous section, with the exception that the recording started after a few minutes in order to ensure that the flow reached steady state conditions at the $Re_{i}$ of interest. Since turbulence was sustained in these measurements, the recording time was set from 90 s at the largest $Re_{i}$ to 15 min at $Re_{i}=525$ (corresponding to $1.4\times 10^{6}$ advective units), which was the lowest $Re_{i}$ at which turbulence was sustained. In general, the observation time was increased, as the critical point was approached (in order to account for the expected critical slowing down). Note that the lids were stationary in these experiments (as for the results shown in Figure 1), which slightly stabilized turbulence when compared to the lifetime measurements with rotating lids discussed in the previous section; with stationary lids turbulence was sustained for $Re_{i}\geq 525$, whereas with rotating lids transient turbulence was found up to $Re_{i}=532$.

As shown in Figure 6a, the spatio-temporal dynamics of turbulent patterns at $Re_{i}=525$ is very rich. Oftentimes a single turbulent stripe spanning the whole system in the axial direction was observed. This then receded and eventually split into two or more arms, one of which would survive and extend to fill the system axially again. Only a slight increase of $Re_{i}$ to 532 was sufficient to almost triple the turbulent fraction, which is reflected by the persistence of more than two turbulent spiral arms (in average) as shown in Figure 6b.

The retrieved turbulent fractions from all measurements are plotted in Figure 7. The minimum measured turbulent fraction is about five times smaller than in previous plane Couette experiments [8], and the maximum observation time in advective units is about 30% longer. The turbulent fraction increases continuously with increasing $Re_{i}$ from its minimum value of about 7% ($Re_{i}=525$) to more than 50%, suggesting a second-order phase transition. The scaling of the turbulent fraction in the vicinity of the critical point is consistent with that expected from directed percolation in two dimensions, $T_{f}=a{\left(Re_{i}-Re_{i,c}\right)}^{\beta }$, where β=0.583, $Re_{i,c}$ is the critical Reynolds number and *a* is a proportionality constant. A least-square fit of this function to the data close to the critical point ($524<Re_{i}<540$) yields a=0.0667 and $Re_{i,c}=524.1$ and approximates very well the data (see the black line in Figure 7). However, measurements closer to the critical point (including a direct determination of the critical point itself) would be necessary to test the robustness and accuracy of this fit. For example, if the function above is fitted with a free exponent, then a=0.0493, $Re_{i,c}=523.5$ and β=0.703 is obtained. Finally, we stress that our system is too small to accurately determine critical exponents. Studies of quasi-one-dimensional Couette flow [26] and of quasi-two-dimensional Waleffe flow [28] show that determining the critical exponents requires a considerably larger system size. Indeed the observed interactions of the stripes with the axially bounding lids demonstrate that the the axial aspect ratio may be insufficient to probe the question of whether transition to turbulence in quasi-two-dimensional Couette flow falls into the directed percolation universality class.

## 4. Discussion

We investigated transient turbulence and the transition to sustained turbulence in a high-radius-ratio Taylor–Couette experiment. The presented lifetime measurements confirm the transient nature of turbulent stripes and show that their decay is memoryless in agreement with the study by Borrero et al. [16] for a smaller Taylor–Couette setup and more generally with transitional turbulence in other shear flows. At lower Reynolds numbers the lifetimes are shorter than the equilibrium time of the flow to adapt to the reduction in Reynolds number, but distributions remain exponential unlike in pipe flow where at low Reynolds numbers the tails deviated from exponential [14]. Our system area is more than 10 times larger than previous Couette and Taylor–Couette experiments, which enables us to approach the critical point much closer without suffering from finite size effects. Whereas such studies in smaller aspect ratio Couette flow had suggested a discontinuous drop form considerably larger turbulent fractions in our case the scaling is continuous, consistent with a second-order phase transition. Our observation of a continuous phase transition is also in line with recent studies of Waleffe flow [28] and of channel flow (see Figure 9a of [46]). An even closer approach to the critical point also leads to a sudden drop in turbulent fraction in the present case. As the critical point is approached length scales diverge and once typical laminar gap sizes exceed the system size the flow relaminarizes. Finite size effects can therefore be mistaken for a discontinuous transition. To resolve this question and to potentially obtain critical exponents, would require an even larger system size which sets a challenge for future experiments. Because of the long laminar gaps separating stripes in the vicinity of the critical point, and of the results of simulations and experiments of quasi-one-dimensional Couette flow [26,38] and Waleffe flow [28], we estimate that order of 1000 gap width are needed in the azimuthal and axial directions to probe for scale invariant flow patterns sufficiently close to the critical point. Such a study would however require cylinders manufactured to considerably higher precision than the already very precise ones used in the present study.

## Figures and Tables

**Table 1:** Summary of experiments (first four rows) and direct numerical simulations (last four rows) of plane Couette and Taylor–Couette flows in the sub-critical regime. Only published works in which lifetimes were determined statistically and/or the turbulent fraction close to onset was measured are listed. The systems investigated by Lemoult et al. [26] and Shi et al. [38] are quasi-one-dimensional (strongly confined in the spanwise direction). In their experiments and DNS the minimum measurable turbulent fraction is constrained by the streamwise length (instead of the area).

Reference	System	Streamwise Length	Spanwise Length	Area
Bottin and Chatté [8]	pCf (η=1)	190*d*	35*d*	6650$d^{2}$
Borrero et al. [16]	TCf (η=0.9)	55*d*	34*d*	1870$d^{2}$
Lemoult et al. [26]	TCf (η=0.998)	2750*d*	8*d*	———
This work	TCf (η=0.98)	311*d*	263*d*	81,793$d^{2}$
Duguet et al. [27]	pCf (η=1)	400*d*	178*d*	71,200$d^{2}$
Shi et al. [26]	TCf (η=0.993)	480*d*	5*d*	———
Lemoult et al. [26]	TCf (η=0.993)	960*d*	5*d*	———
Chantry et al. [28]	Waleffe flow	1280*d*	1280*d*	1,638,400$d^{2}$

## Data Availability

Data sharing not applicable.
